# The H2A.Z-KDM1A complex promotes tumorigenesis by localizing in the nucleus to promote SFRP1 promoter methylation in cholangiocarcinoma cells

**DOI:** 10.1186/s12885-022-10279-y

**Published:** 2022-11-11

**Authors:** Qi Wang, Yongqiang Qi, Fei Xiong, Da Wang, Bing Wang, Yongjun Chen

**Affiliations:** 1 Department of Biliary-Pancreatic Surgery, Tongji Hospital, Tongji Medical College, Huazhong University of Science and Technology, No. 1095 Jiefang Road, Wuhan, 430074 Hubei Province China; 2 Department of General Surgery, The Fifth People’s Hospital of Shanghai, Fudan University, Shanghai, China

**Keywords:** H2A.Z, KDM1A, SFRP1, Methylation, H3K4me1/2, Intrahepatic cholangiocarcinoma

## Abstract

**Background:**

Intrahepatic cholangiocarcinoma (ICC), originating from the bile ducts, is the second most common primary liver malignancy, and its incidence has recently increased. H2A.Z, a highly conserved H2A variant, is emerging as a key regulatory molecule in cancer. However, its underlying mechanism of action in ICC cells remains unclear.

**Methods:**

Here, we examined the expression of H2A.Z and SFRP1 in normal intrahepatic cholangiocytes, ICC cell lines, ICC tissue microarrays, and fresh specimens. The correlations between H2A.Z or SFRP1 expression and clinical features were analysed. The overall survival rate was analysed based on H2A.Z and SFRP1 expression. Immunoprecipitation was used to analyse the recruitment of KDM1A, and ChIP sequencing and BSP were used to analyse the enrichment of methylation-related molecules such as H3K4me1 and H3K4me2 in the SFRP1 promoter and reveal the underlying mechanisms. Knockdown and rescue experiments were used to determine the potential mechanism by which H2A.Z and SFRP1 promote tumorigenesis in vitro.

**Results:**

We showed that upregulation of H2A.Z expression is linked to downregulation of SFRP1 expression in ICC tissues and poor overall survival in patients with ICC. H2A.Z interacted with KDM1A in the nucleus to bind to the -151 ~ -136 bp region upstream of the SFRP1 promoter to increase its demethylation in ICC cells. Functionally, H2A.Z silencing inhibited the proliferation and invasion of ICC cells, and these effects were mitigated by SFRP1 silencing in ICC cells.

**Conclusions:**

Our findings reveal that H2A.Z inhibits SFRP1 expression through chromatin modification in the context of ICC by forming a complex with KDM1A in the nucleus.

**Supplementary Information:**

The online version contains supplementary material available at 10.1186/s12885-022-10279-y.

## Background

Intrahepatic cholangiocarcinoma (ICC), originating from the bile ducts, is the second most common primary liver malignancy, and its incidence has recently increased [1, 2]. Currently, although there are a variety of treatment strategies, such as selective resection and hepatic arterial infusion chemotherapy, their efficacy is limited. Therefore, it is of great clinical significance for the management of patients with ICC to understand the molecular pathogenesis of ICC and find new therapeutic targets [3, 4].

Epigenetic modifications are crucial for physiological development and homeostatic gene expression in eukaryotes [5] and are required for a variety of biological processes ranging from gene expression to disease pathogenesis [6]. DNA methylation, histone modifications, and posttranslational modifications (PTMs) represent epigenetic alterations that may, individually or in combination, modify chromatin structure and gene activity by promoting either activation or repression of genes depending on the type of regulator [7]. DNA methylation is a widely studied epigenetic mechanism. Aberrant DNA methylation is an early and stable event in carcinogenesis. The identification of specific DNA methylation patterns has the potential to be diagnostic markers for early detection and lead to the development of therapeutic modalities [8]. Genome-wide hypomethylation and specific CpG island hypermethylation are the most common changes in tumour-related molecular biology [9].

H2A.Z is a highly conserved H2A variant, with only 60% identity to canonical H2A, and is expressed and incorporated into chromatin throughout the cell cycle [10]. Although somewhat confused by species-specific functions and context dependencies, the role of H2A.Z in transcriptional regulation has been demonstrated [11]. H2A.Z is enriched at gene promoters, as well as other regulatory regions, generally exerting a positive effect on transcription [12]. Several studies have shown that H2A.Z is overexpressed in breast, prostate, and bladder cancers during tumorigenesis, in some cases, regulates proliferation [13]. However, its functional role in cholangiocarcinoma is unclear.

KDM1A, the first demethylase to be identified, was considered a nuclear protein [14]. KDM1A can remove mono- and dimethylation from histone H3 at lysine 4 (H3K4me1/2), modifications that are associated with active promoters and either latent or active enhancers [15]. Researchers have also found that KDM1A appears to function as a coactivator and mediate the removal of the repressive mark H3K9me2 [16]. Therefore, using the STRING database, we predicted the functional protein association networks of H2A.Z. Interestingly, we found that H2A.Z may form a complex with KDM1A. The results were visualized with Cytoscape.

Secreted frizzled-related protein 1 (SFRP1) is a gene that belongs to the secreted glycoprotein SFRP family. It has been identified as a tumour suppressor because it is lost in expression in many human cancers [17]. SFRP1 is homologous to the putative Wnt-binding site of frizzled (Fz) receptors. Therefore, SFRP1 can act as a modulator of the Wnt signaling pathway [18]. Silencing of SFRP1 may cause dysregulation of cell proliferation, migration, and invasion, which lead to tumorigenesis, disease progression, resistance to treatment, and a poorer prognosis [17]. Endogenous SFRP1 expression increases in a dose-dependent manner after demethylating treatment, signifying DNA methylation as the main mechanism that is responsible for the silencing of SFRP1[19].

It has been shown that H2A is involved in the integration of the Wnt pathway [20], promotes the activation of the Wnt pathway together with Acidic nuclear phosphoprotein 32 family member E (ANP32E) [21], and participates in the regulation of the Wnt pathway together with SOX2 [22]. Given that both H2A and SFRP1 are closely related to the Wnt pathway, we explored whether there is a potential link between H2A and SFRP1. In this study, we identified a new mechanism by which H2A.Z inhibits SFRP1 expression by forming a complex with KDM1A in the nucleus of ICC cells through binding to the -151 ~ -136 bp region of upstream and demethylating the SFRP1 promoter, thus promoting tumorigenesis in the context of ICC.

## Methods

### Microarrays

Two microarrays containing the mRNA sequencing data of cholangiocarcinoma tissue (GSE32879 and GSE76297) and two microarrays containing methylation data of cholangiocarcinoma tissue (GSE38860 and GSE49656) were downloaded from the Gene Expression Omnibus database (GEO; https://www.ncbi.nlm.nih.gov/geo/) [23]. The data were normalized according to the description provided by the uploaders, which is available in the database. The details of the microarrays are shown in Table S1.

### Antibodies

Antibodies, including rabbit anti-H2A.Z (CST, Leiden, the Netherlands, 50722S), anti-H3K4me1 (CST, 5326S), rabbit polyclonal anti-Histone 3 (Proteintech, Chicago, USA, 17,168–1-AP), anti-H3K4me2 (CST, 9725S), anti-KDM1A (CST, 2139S), anti-SFRP1 (CST, 3534S), goat polyclonal anti-HP1α (Abcam, MA, USA, ab77256), mouse monoclonal anti-GST (Proteintech, 66,001–1-Ig), mouse monoclonal anti-GAPDH (Boster, Wuhan, China, BM1623) and IgG from healthy animals (Beyotime, Shanghai, China, A7007) were obtained from different companies.

Twenty-five pairs of human ICC and paracancerous tissues were collected from the Department of Biliary-Pancreatic Surgery at Tongji Hospital of Huazhong University of Science and Technology. With the written informed consent of individual patients, the experimental protocol was approved by the Tongji Hospital Ethics Committee.

### Cell culture and transfection

Human HuccT-1, HCCC-9810 and RBE ICC cells, as well as HIBEpic cholangiocytes, were cultured in RPMI 1640 medium supplemented with 10% foetal bovine serum (FBS) in a humidified incubator with 5% CO2 at 37 °C. HEK293T cells were cultured in DMEM/high glucose medium supplemented with 10% FBS. The control lentivirus (LV-siR-negative control, abbreviated LV-siRNA-NC), the lentivirus for expressing KDM1A-specific siRNA, and the lentivirus for expressing H2A.Z-specific shRNA were produced by GeneChem (Shanghai, China). The SFRP1-specific small interfering RNA was constructed and synthesized by RiboBio (Guangzhou, China). The cDNA fragment of the targeted gene was extracted from HEK293T cells, amplified by PCR and then inserted into the lentiviral vector pGC-LV carrying a red fluorescent protein (mCherry) tag. The final lentiviral construct, U6-MCS-Ubi-EGFP, was confirmed by DNA sequencing. After transfection, lentivirus was produced in HEK293T cells. To generate stable cell lines, separate groups of cells were transduced with lentiviruses (0.3 ml, 107 TU/ml) in complete medium containing polybrene for 24 h and then cultured in fresh RPMI 1640 medium for another 48 h [24].

### RNA extraction and quantitative real-time PCR

Total RNA was extracted from the various cell groups using TRIzol reagent (Invitrogen, California, USA), and each RNA sample (400 ng) was reverse transcribed into cDNA using a PrimeScript RT Reagent Kit (Takara, Dalian, China), as directed by the manufacturer. The relative levels of targeted gene mRNA transcripts to the control GAPDH were measured by quantitative real-time PCR using ChamQ Universal SYBR qPCR Master Mix (Vazyme, Nanjing, China) and specific primers in the iQ5 quantitative PCR detection system (Bio-Rad, Richmond, CA, USA) [23]. The sequences of the primers were as follows: SFRP1-F, 5’-GAGCCGGTCATGCAGTTCTT-3’ and SFRP1-R, 5’-CGTTGTCACAGGGAGGACAC-3’; H2A.Z-F, 5’-GGCAGGAAATGCATCAAAAG-3’ and H2A.Z-R, 5’-TGGATGTGTGGAATGACACC-3’; GAPDH-F, 5’-TTTGTCAAGCTCATTTCCTGG-3’ and GAPDH-R, 5’-TGATGGTACATGACAAGGTGC-3’. The data were analysed using IQ5 software.

### Western blotting

Cells were harvested, lysed in lysis buffer containing protease inhibitors and phosphatase inhibitors (Roche, Basel, Switzerland), and centrifuged. Proteins in the cell lysates (50 g) were separated by 12% sodium dodecyl sulfate–polyacrylamide gel electrophoresis (SDS–PAGE) and transferred onto polyvinylidene fluoride (PVDF) membranes after identification and quantification (Millipore, Billerica, MA, USA). After blocking with 5% skim milk powder in TBST, the membranes were incubated with the corresponding antibodies overnight at 4 °C, and the bound antibodies were detected with horseradish peroxidase (HRP)-conjugated secondary antibodies and observed by enhanced chemiluminescence (ECL; Millipore). Relative levels of protein expression were determined by densitometric analysis using ImageJ software [25].

### Immunofluorescence

LV-siRNA-NC and LV-siR-H2A.Z HuccT-1 and RBE cells (10^5^ cells/well) were cultured on glass coverslips for 48 h, fixed with 4% paraformaldehyde and permeabilized with 0.1% Triton X-100. The cells were stained with PE-anti-H2A.Z or isotype control antibodies. After rinsing, the cover slips were mounted on the slides with mounting medium containing DAPI, and fluorescence signals were detected with a fluorescence microscope.

### Coimmunoprecipitation

The functional protein association network of H2A.Z was predicted with the STRING database (https://cn.string-db.org/). The results were visualized with Cytoscape. The potential interactions among H2A.Z and KDM1A/H3K4me1/H3K4me2 were identified by coimmunoprecipitation. Briefly, cells were lysed in IP lysis buffer (50 mM Tris–HCl (pH 7.4), 150 mM NaCl, 1 mM EDTA, 1% NP-40 and 10% glycerin) containing protease inhibitors and phosphatase inhibitors (Roche). Then, separate cell lysates were incubated with 2.5 μg of anti-H2A.Z/KDM1A/H3K4me1/H3K4me2 antibodies and 20 μl of Protein A + G agarose beads (Beyotime) overnight at 4 °C with gentle shaking. After being washed, the bound proteins were eluted and characterized by immunoblotting using anti-H2A.Z, anti-H2A.Z, anti-H3K4me1, and anti-H3K4me2 antibodies. For western blot analysis, SDS–PAGE was performed, and the proteins were transferred to PVDF membranes (ISEQ00010, Millipore, Merck KGaA, Germany). After blocking with 5% nonfat milk, the membranes were incubated with primary antibodies at 4 °C overnight. The membranes were then incubated with secondary antibodies, namely, horseradish peroxidase-conjugated goat anti-mouse IgG and goat anti-rabbit IgG (Santa Cruz Biotechnology, CA, USA; as the negative control), at room temperature for 1 h, after which the immunoreactive bands were visualized using the image analysis software ImageJ (National Institutes of Health, Bethesda, MD, USA) [24].

### Immunohistochemistry (IHC)

An ICC tissue microarray containing 25 ICC tissues and paired nontumor tissues was analysed. All samples were dewaxed, rehydrated and subjected to antigen repair. The sections were incubated with the anti-H2A.Z (1:500) or anti-SFRP1 (1:500) antibody overnight at 4 °C, and after washing, bound antibodies were detected by incubation with biotinylated secondary antibodies for 30 min at 37 °C and then visualized with streptavidin–biotin-peroxidase and DAB. Haematoxylin was used to counterstain the sections. The percentage and intensity of positive staining in the cells were used for semiquantitative scoring of immunohistochemical signals. 0: < 5% positive cells; 1: 5% to 24% positive cells; 2: 25% to 49% positive cells; 3: 50% to 74% positive cells; and 4: ≥ 75% positive cells. For intensity scoring, an absence of staining was scored as 0, weak staining as 1, moderate staining as 2 and strong staining as 3. The final staining index was equal to the percentage of positive staining score multiplied by the intensity score and was categorized as follows: no staining, 0–1; weak staining, 2–4; moderate staining, 5–8; and strong staining, 9–12. The immunostaining was blindly scored by 2 pathologists.

### Chromatin immunoprecipitation (ChIP)

Approximately 10^7^ cells were fixed with 1% formaldehyde to crosslink endogenous protein with DNA. After ultrasonication, the DNA/protein complexes were immunoprecipitated with primary antibodies and protein A/G agarose beads, with IgG used as the negative control. Quantitative real-time PCR was used to examine the precipitated DNA and input DNA using specific primers. The sequences of the primers were as follows: SFRP1-F, 5’-GTTGCAGTCAGCGGAGAT-3’ and SFRP1-R, 5’-G GAGCCTGGATCATACTTGC-3’; β-actin-F, 5’-CCCTCCTCCTCTTCCTCAATCT-3’ and β-actin-R, 5’-AACGGCGCACGCTGATT-3’.

### Bisulfite sequencing PCR (BSP)

The BSP primers were designed using the online program MethPrimer (http://www.urogene.org/methprimer). The sequences of the primers used for BSP were BSP-SFRP1-F: 5’-TTGGGAGTTGATTGGTTG-3’ and BSP-SFRP1-R: 5’-CTTCCAAAAACCTCCAAAAA-3’. For BSP, ddH2O, 10 × PCR buffer, dNTP mix, PCR primers, rTaq, and bisulfite-converted DNA samples were mixed in a final volume of 25 μl. The PCR products were inserted into the pMD19-T vector. Ten clones were randomly selected from each group and sequenced by OeBiotech (Shanghai, China).

### Cell proliferation assay

Cells of different groups (5 × 10^3^ cells/well) were cultured with 10% FBS RPMI 1640 medium in 96-well plates for 48 h. Cell Counting Kit-8 (CCK-8) reagent (40203ES60, Yeasen, Shanghai, China) was added to individual wells during the last hour of culture. The absorbance (OD) value, representing cell proliferation, was measured at 450 nm in a microplate reader (Bio-Rad).

### Wound healing and Transwell invasion assays

Cell monolayers in individual wells were scraped with a sterile pipette tip (100 μl) after the different groups of ICC cells (1 × 10^6^ cells/well) were grown to 90% confluence. The cells were incubated for 24 h and photographed with a digital camera (Leica, Heerburg, Germany) at 0 h and 24 h after wounding. The distance of cell migration into the denuded area was used to determine the degree of wound healing [24].

The different groups of ICC cells (1 × 10^4^ cells/well) were seeded in the upper chambers of 24-well Transwell plates containing Matrigel-coated membranes (BD Biosciences, San Jose, CA, USA). Complete medium was added to the bottom chambers. After 36 h of incubation, uninvaded cells were removed from the top surface of the membrane in the upper chamber with a cotton swab. Invaded cells on the underside of the membrane in the upper chamber were fixed with 4% paraformaldehyde, stained with 1% crystal violet and counted under a microscope in a blinded manner [24].

### Mouse model

Twenty female nude mice (5 weeks old) were purchased from Charles River, Beijing, China. The mice were evenly and randomly divided into four groups. Each mouse was injected subcutaneously with 2 × 10^6^ HuccT-1 cells in the axillary cavity. Then, the mice were euthanized through cervical dislocation after three weeks. The tumours were excised, and the volumes were calculated. The tumour volumes were calculated with the following equation: tumour volume (mm3) = 0.5 × (length × width^2^). This study was approved by the Animal Experimentation Ethics Committee of Tongji Medical College. Animal care and experimental procedures were performed according to the criteria outlined in the current NIH guidelines and ARRIVE guidelines.

### Luciferase reporter assay

HEK293T cells (1 × 10^5^ cells/well) were grown to 50% confluence in 96-well plates and cotransfected in triplicate with 0.5 µg of SFRP1-pGL3 (or pGL3), 0.1 µg of pDNA-H2A.Z (or pDNA) and the Renilla luciferase plasmid using Lipofectamine 2000 (Invitrogen). After 24 h of transfection, the luciferase activity in different groups of cells was measured with a luminometer (Lumat LB9507, Berthold, Germany) and the Dual-Luciferase Reporter Assay System (Promega, Wisconsin, USA). For each individual analysis, firefly luciferase was used as the reporter gene and Renilla luciferase as the normalization control.

### Statistical analysis

All data are expressed as the means ± standard errors of the means (SEMs). Two-tailed Student’s t test was used to evaluate differences between two independent samples, and two-tailed ANOVA was used to evaluate differences among more than two groups using GraphPad Prism 5.0 (GraphPad Prism Software Inc., San Diego, CA, USA). A *P* value of < 0.05 was considered to be statistically significant.

## Results

### H2A.Z expression is upregulated in ICC tissues and associated with downregulated SFRP1 expression and poor prognosis in patients with ICC

**Fig. 1:**
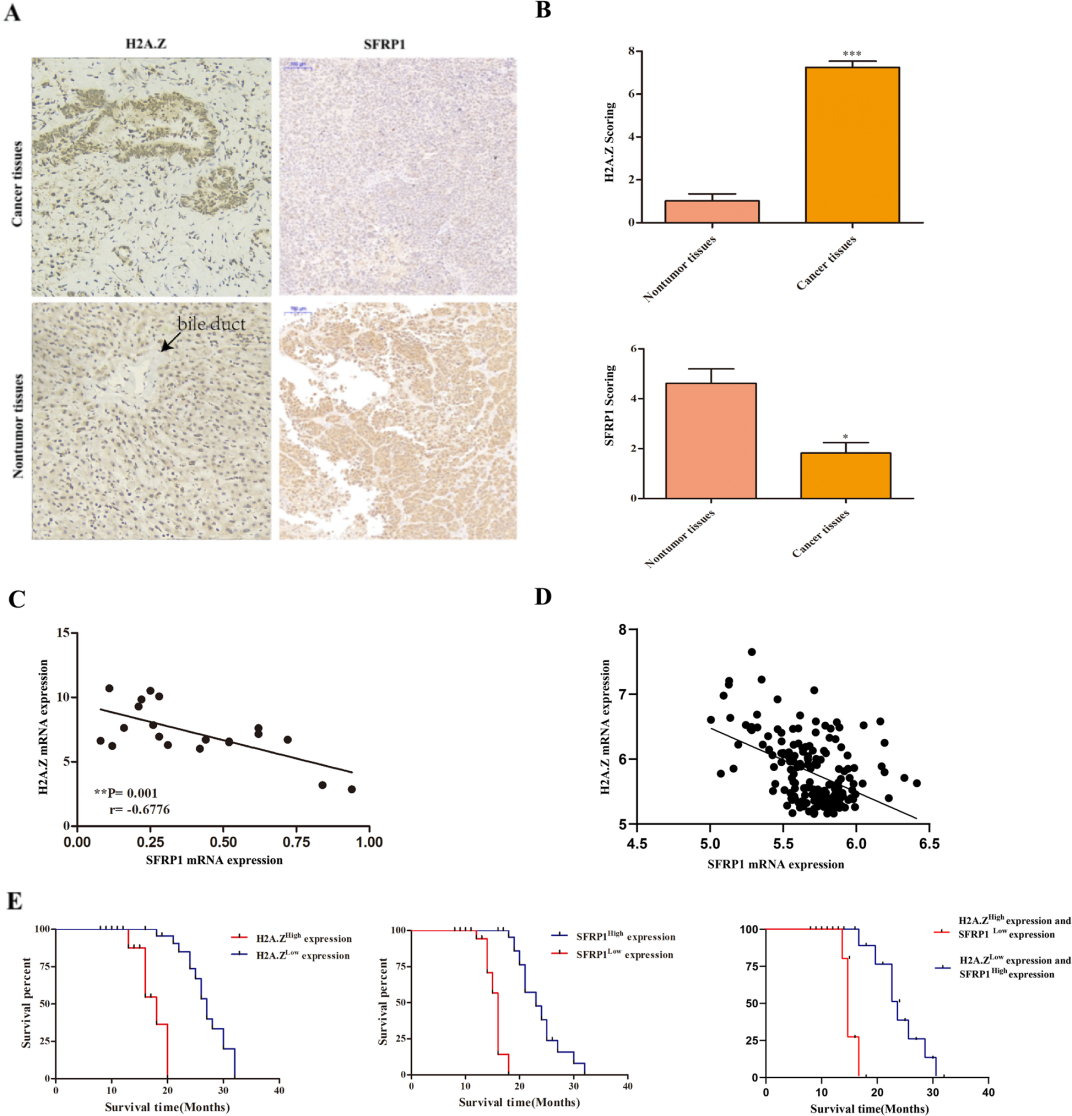
Upregulation of H2A.Z is associated with downregulation of SFRP1 and poor survival. **A-B** The levels of H2A.Z and SFRP1 in 25 primary ICC and paired adjacent nontumor tissues were examined by IHC. Scale bar, 100 μm. **B** The expression levels of H2A.Z (upper panel) and SFRP1 (lower panel) were semiquantitatively analysed. **C** The mRNA transcript levels of H2A.Z and SFRP1 were measured by quantitative RT–PCR in 20 pairs of ICC and paracancerous tissues, and Pearson correlation coefficients were calculated (*r* =  − 0.6776, **p = 0.001). **D** The mRNA transcript levels of H2A.Z and SFRP1 were quoted from the GSE76297, and Pearson correlation coefficients were calculated (*r* =  − 0.04623, ****p = 0.001). **E** Kaplan–Meier analysis of overall survival in 50 ICC patients with higher H2A.Z (*n* = 25) and lower SFRP1 (*n* = 25) expression vs. patients with lower H2A.Z (*n* = 25) and higher SFRP1 (*n* = 25) expression. The data shown are representative images, individual mean values or the mean ± SD of each group in three independent experiments. *p < 0.05, **p < 0.01, ***p < 0.001

To understand the potential link between H2A.Z and SFRP1 during the development and progression of ICC, the levels of H2A.Z and SFRP1 expression in 25 primary ICC and paired adjacent nontumor tissues were measured by immunohistochemistry (IHC). As shown in Fig. 1A, H2A.Z expression was mainly localized in the nucleus in ICC cells. Compared with that in the noncancerous bile duct epithelium, the H2A.Z expression was upregulated and SFRP1 expression was downregulated in ICC tissues. Semiquantitative analysis indicated that the levels of H2A.Z expression in the ICC tissues were significantly higher than those in the nontumor tissues (7.15 ± 0.43 vs. 1.52 ± 0.28, ***p < 0.001), while the expression level of SFRP1 was significantly lower in the ICC tissues than in the nontumour tissues (1.82 ± 0.58 vs. 4.85 ± 0.30, *p < 0.05; Fig. 1B). We also found that H2A.Z was upregulated and SFRP1 was downregulated in ICC tissues according to the data in GSE32879 and GSE76297 (Fig. S1A, B). Further quantitative RT–PCR of 20 paired ICC and adjacent nontumor tissue samples revealed that 18 of the 20 pairs of samples had higher levels of H2A.Z expression and lower levels of SFRP1 expression in the ICC tissues, with a Pearson correlation coefficient of -0.6776 (**P = 0.001, Fig. 1C). The levels of H2A.Z expression and SFRP1 expression in the ICC tissues according to the data in GSE 76,297 also have the same correlation, with a Pearson correlation coefficient of -0.4623 (****P = 0.001, Fig. 1D). Western blot analysis showed that the relative expression level of SFRP1 in the tumour samples was lower than that in the nontumor tissues, while the relative expression level of H2A.Z in the tumour tissues was higher than that in the nontumor tissues (Fig. S1C). The above results suggest that the expression of H2A.Z is opposite to that of SFPR1 in ICC, but do not yet indicate a negative correlation between the two expression levels.

**Fig. 2:**
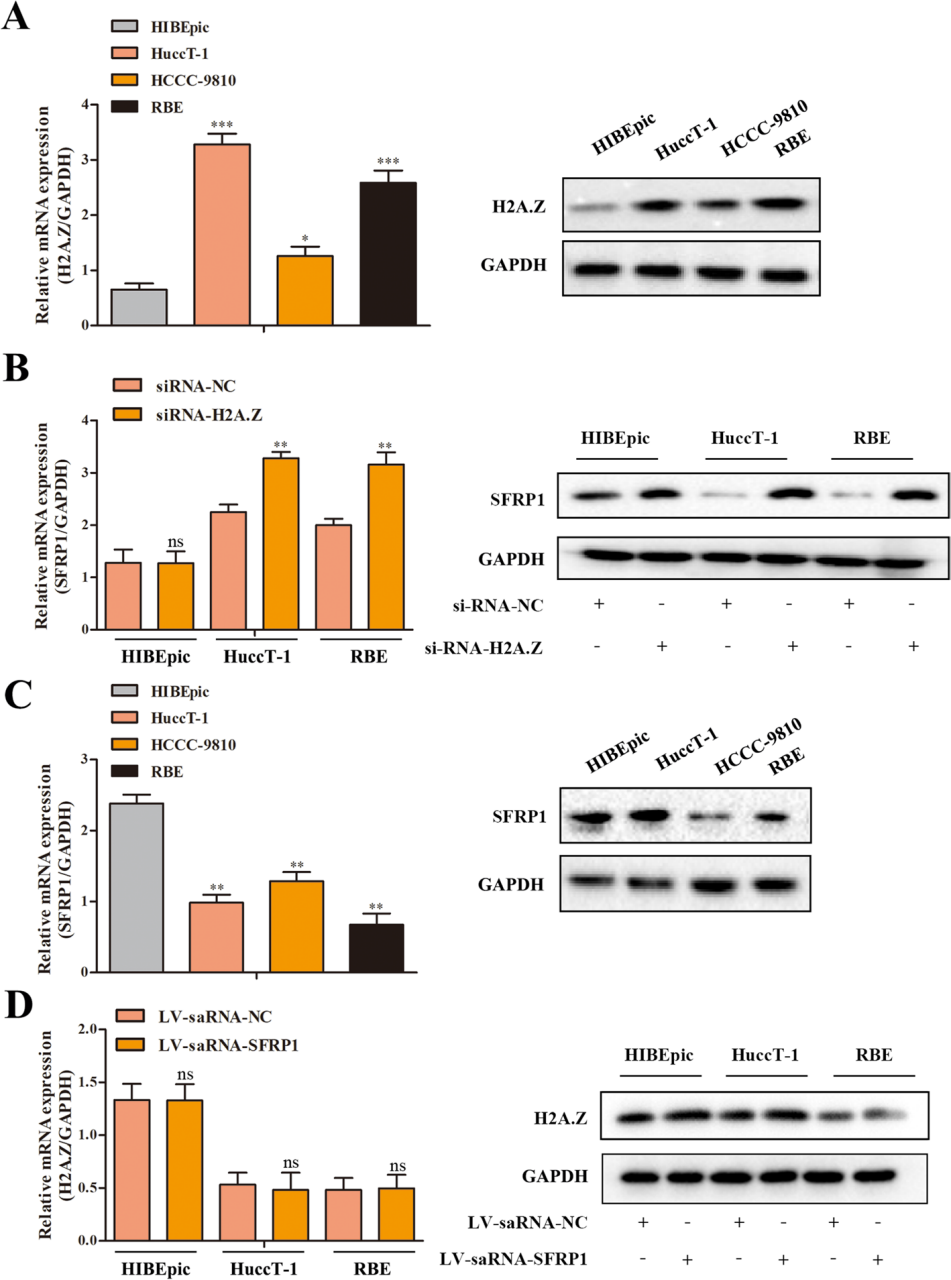
H2A.Z silencing enhances SFRP1 expression in ICC cells. **A**, **C** The relative levels of H2A.Z and SFRP1 expression were determined in ICC and nontumor HIBEpic cells by quantitative RT–PCR and Western blotting. **B** H2A.Z silencing and related changes in SFRP1 expression were demonstrated in HuccT-1, RBE and HIBEpic cells by quantitative RT–PCR and Western blotting. **D** SFRP1 overexpression and H2A.Z expression were demonstrated in HuccT-1, RBE and HIBEpic cells by quantitative RT–PCR and Western blotting. The data shown are representative images, individual mean values or the mean ± SD of each group in three independent experiments. *p < 0.05, **p < 0.01, ***p < 0.001

Then, we investigated whether the expression of H2A.Z and SFRP1 was associated with clinicopathological characteristics in 50 patients with ICC. We found that overexpression of H2A.Z was associated with pathological grade, lymph node metastasis and distant metastasis (*P = 0.021, *P = 0.028, and *P = 0.047, respectively) but not to age or sex (Table 1). More importantly, stratification of the patients according to the high (or positive) or low (or negative) expression of H2A.Z or SFRP1 showed that the patients with higher H2A.Z or lower SFRP1 expression had significantly shorter overall survival times than those with lower H2A.Z or higher SFRP1 expression in this population. At the same time, compared to the patients with simultaneous lower H2A.Z and higher SFRP1 expression, those with simultaneous higher H2A.Z and lower SFRP1 expression had shorter overall survival times (Fig. 1E). Therefore, downregulated SFRP1 expression and upregulated H2A.Z expression in ICC has a worse prognosis for patients with ICC.

**Table 1:** Associations between H2A.Z expression and clinicopathologic characteristics

Characteristic	Case (Number)	H2A.Z expression		*P* value
		Low expression (*N* = 23)	High expression (*N* = 27)	
Age (years)	≤ 55 (25)	10	15	0.395
	> 55 (25)	13	12	
Sex	Male (22)	12	10	0.283
	Female (28)	11	17	
Pathological grade	I + II (12)	9	3	*0.021*
	III + IV (38)	14	24	
Lymph node metastasis	Negative (20)	13	7	*0.028*
	Positive (30)	10	20	
Distant metastasis	M0 (25)	15	10	*0.047*
	M1 (25)	8	17	

The italicized values indicate *p* < 0.05

### Knockdown of H2A.Z enhances SFRP1 expression in ICC cells

To understand whether there is a regulatory relationship between H2A.Z and SFRP1, we first determined the relative levels of H2A.Z expression in HuccT-1, HCCC-9810 and RBE ICC cells as well as the nontumor cholangiocyte line HIBEpic by quantitative RT–PCR and Western blot analysis. As shown in Fig. 2A, the relative H2A.Z expression levels in the ICC cells were significantly higher than that in nontumor HIBEpic cells. Interestingly, we also found that SFRP1 expression was lower in the ICC cell lines than in HIBEpic cells (Fig. 2C).

Next, we employed lentiviral siRNA vectors to silence H2A.Z expression in HuccT-1, RBE and HIBEpic cells (Fig. 2B). We found that H2A.Z silencing increased the relative level of SFRP1 expression in both HuccT-1 and RBE cells but made no apparent difference in HIBEpic cells. Interestingly, we generated SFRP1-overexpressing HuccT-1, RBE and HIBEpic cells using the saRNA technique (Fig. 2D). Induction of SFRP1 overexpression did not reduce H2A.Z expression in HuccT-1, RBE or HIBEpic cells at either the mRNA or the protein level. Together, these results suggested that SFRP1 is a downstream target gene of H2A.Z and has no significant regulatory effect on H2A.Z.

### H2A.Z is predominantly localized in the nucleus to regulate SFRP1 expression through the KDM1A/H3K4me1/H3K4me2 complex in ICC cells

**Fig. 3:**
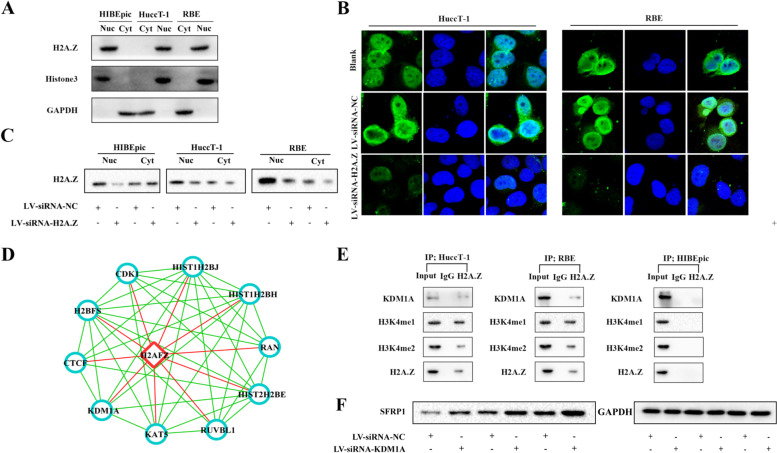
H2A.Z forms a complex with KDM1A, H3K4me1 and H3K4me2. **A** Western blot analysis revealed that H2A.Z is mainly expressed in the nucleus in HuccT-1 and RBE cells. **B** Immunofluorescence (IF) analysis of the nuclear localization of H2A.Z in HuccT-1 and RBE cells (400 × magnification). **C** Western blot analysis revealed that H2A.Z is mainly expressed in the nucleus in HuccT-1 and RBE cells with H2A.Z silencing. **E** Endogenous KDM1A, H3K4me1 and H3K4me2 were precipitated with the anti-H2A.Z antibody in HuccT-1, RBE and HIBEpic cells. **F** Western blot analysis revealed that KDM1A silencing can increase the relative level of SFRP1 expression

To understand the regulatory role of H2A.Z in SFRP1 expression, we determined the relative levels of H2A.Z in the cytoplasm and nucleus using lentiviral siRNA to silence H2A.Z expression in HuccT-1, RBE and HIBEpic cells and found that H2A.Z was predominantly localized in the nucleus in HuccT-1 and RBE cells, while there was no apparent difference in the amounts of H2A.Z in the cytoplasm and the nucleus in HIBEpic cells (Fig. 3A and C). Staining with a FITC-conjugated anti-H2A.Z antibody and DAPI also showed that H2A.Z was mainly localized in the nucleus in HuccT-1 and RBE cells, but significantly reduced fluorescence signals were detected in H2A.Z-silenced HuccT-1 and RBE cells (Fig. 3B).

Some previous studies have demonstrated that H2A.Z was shown to interact with components of complexes involved in a multitude of biological processes, such as DNA damage repair [26], gene activation [27], gene repression [28], various transcription factors [29–31], and chromatin remodeling [32]; however, the underlying mechanism of action remains unclear in ICC. Using the STRING database, we identified potential molecules that interact with H2A.Z (Fig. 3D), and we hypothesized that H2A.Z might form a complex with the histone demethylase KDM1A in ICC cells and, as a result, regulate SFRP1 expression.

To test this hypothesis, we performed coimmunoprecipitation (Co-IP) and found that KDM1A, H3K4me1, and H3K4me2 were precipitated with the anti-H2A.Z antibody in HuccT-1 and RBE cells, while these related chromatin modification markers were not detected in HIBEpic cells (Fig. 3E). Then, we using lentiviral siRNA to silence KDM1A expression in HuccT-1, RBE, and HIBEpic cells and found that KDM1A silencing increased the relative level of SFRP1 expression in both HuccT-1 and RBE cells (Fig. 3F). These findings indicated that H2A.Z participates in the KDM1A/H3K4me1/H3K4me2 complex to inhibit SFRP1 expression via chromatin modification in ICC cells.

### H2A.Z promotes SFRP1 promoter demethylation in ICC cells

**Fig. 4:**
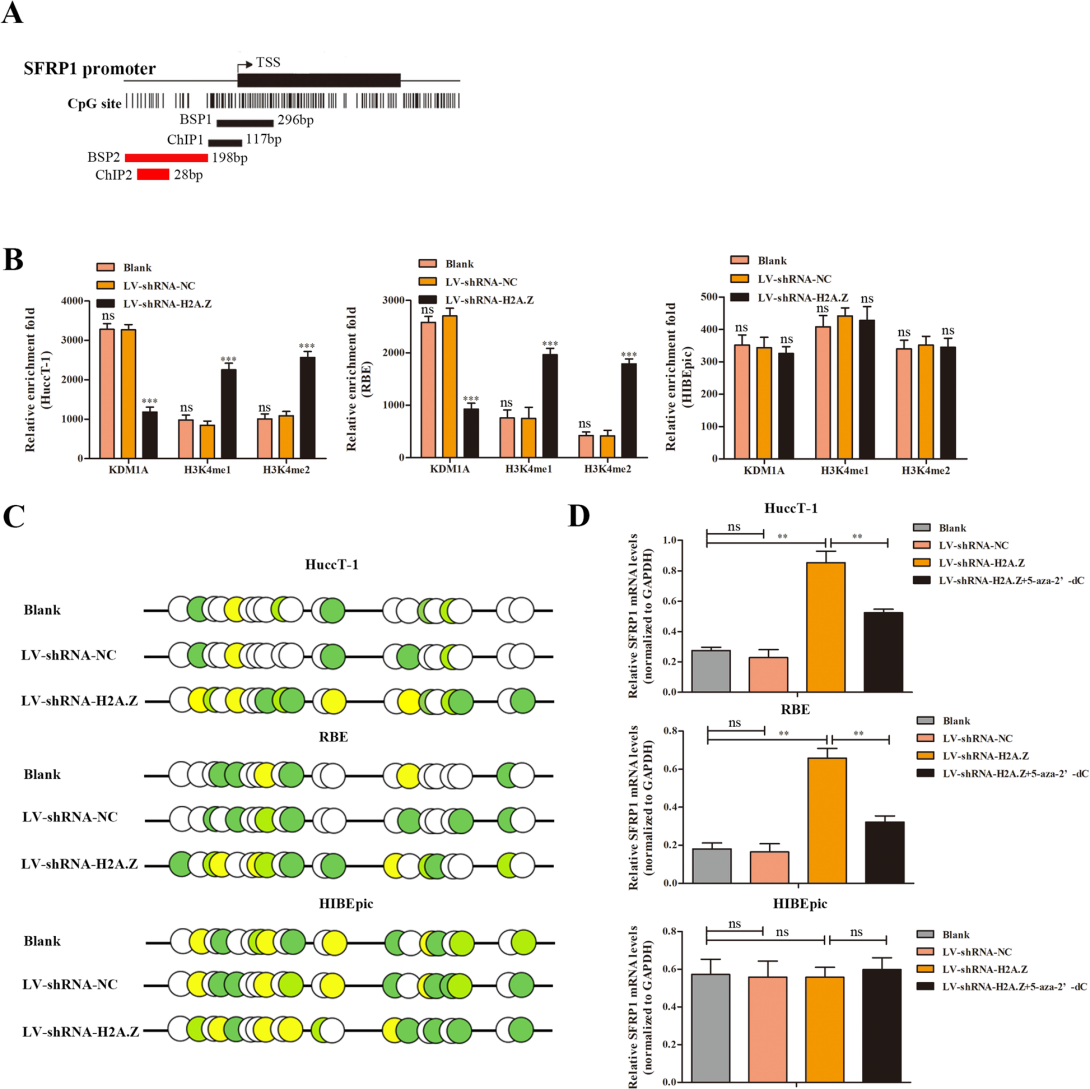
H2A.Z binds to the upstream region of the SFRP1 promoter and reduces SFRP1 expression. **A** Graphical representation of the CpG sites in the SFRP1 promoter. **B** ChIP assay of KDM1A, H3K4me1 and H3K4me2 enrichment in the P3 region upstream of the SFRP1 promoter. IgG was used as the negative control. Relative fold enrichment = [%(ChIP/Input)]/[%(IgG/Input)]. **C** BSP analysis of the CpG site methylation rate. Each dot represents one CpG site. White represents the absence of methylation, and pale green to deep yellow represent increasing methylation rates. **D** Quantitative RT–PCR analysis of the SFRP1 mRNA transcript level relative to the GAPDH mRNA transcript level in the blank control, LV-shRNA-NC, LV-shR-H2A.Z and LV-NC-H2A.Z + 5-aza-dC groups. The data are expressed as the individual mean values or the mean ± SD of each group in three independent experiments. **p < 0.01, ***p < 0.001

Our previous findings indicate that the expression of SFRP1 can be affected by HP1α, which is mainly localized in heterochromatic regions of the genome and binds to histone H3 with Lys9 trimethylation (H3K9me3). As Fig. 4A and Fig. S2A show, the downregulation of HP1α can decrease H3K9me3 enrichment and the DNA methylation rate in the P1 region (-116 ~  + 1 bp) of the SFRP1 promoter, resulting in the increase of SFRP1 expression [26]. In order to explore whether H2A.Z, similar to HP1α, affects SFRP1 expression by influencing the histone methylation level and DNA methylation rate of the SFRP1 promoter. We next determined the region of H2A.Z that binds to the SFRP1 promoter through a ChIP assay. Interestingly, the results demonstrated that H2A. Z specifically interacted with the P3 region (-151 ~ -136 bp) upstream of the SFRP1 promoter (Fig. S2A).

Further ChIP assays indicated that H2A.Z silencing significantly decreased the relative level of KDM1A and increased the levels of H3K4me1 and H3K4me2 in both HuccT-1 and RBE cells but showed no effect on HIBEpic cells (Fig. 4B). The CpG island in the promoter of SFRP1 is hypermethylated in ICC tissues, according to the methylation data in GSE38860 and GSE49656 (Fig. S3A, B) BSP indicated that the percentages of methylated CpG in HuccT-1 and RBE cells transduced with LV-shR-NC and blank control cells were significantly lower than that in LV-shR-H2A.Z-transduced cells, while no difference was found in HIBEpic cells (Fig. 4C). Subsequently, H2A.Z silencing or treatment with 5-azacitidine (5-aza-2’-deoxycytidine) to induce demethylation significantly increased the relative SFRP1 mRNA transcript level relative to those in HuccT-1 and RBE cells transduced with LV-shR-NC and blank control cells (Fig. 4D).

We next determined how H2A.Z regulates SFRP1 promoter-driven luciferase expression in HEK293T cells by performing a dual luciferase assay. A 500 bp fragment upstream of the SFRP1 transcription start site that included the CpG island-enriched region and the P3 region was inserted into the pGL3 plasmid (with the negative control plasmid called pGL3), and this plasmid was cotransfected with the H2A.Z expression plasmid (with the negative control plasmid called pDNA) as well as the Renilla luciferase expression plasmid into HEK293T cells and into HuccT-1 and RBE cells. The results indicated that induction of H2A. Z overexpression significantly reduced the luciferase activity of the SFRP1 promoter that was inserted into the pGL3 plasmid in these cells compared with H2A.Z negative control plasmid-transfected cells (Fig. S2B).

Collectively, our data indicated that H2A.Z was recruited to the P3 region upstream of the SFRP1 promoter and formed a complex with KDM1A to promote the removal of H3K4me1 and H3K4me2 to reduce SFRP1 expression in ICC cells.

### H2A.Z silencing inhibits the proliferation and invasion of ICC cells, and these effects are mitigated by SFRP1 silencing

**Fig. 5:**
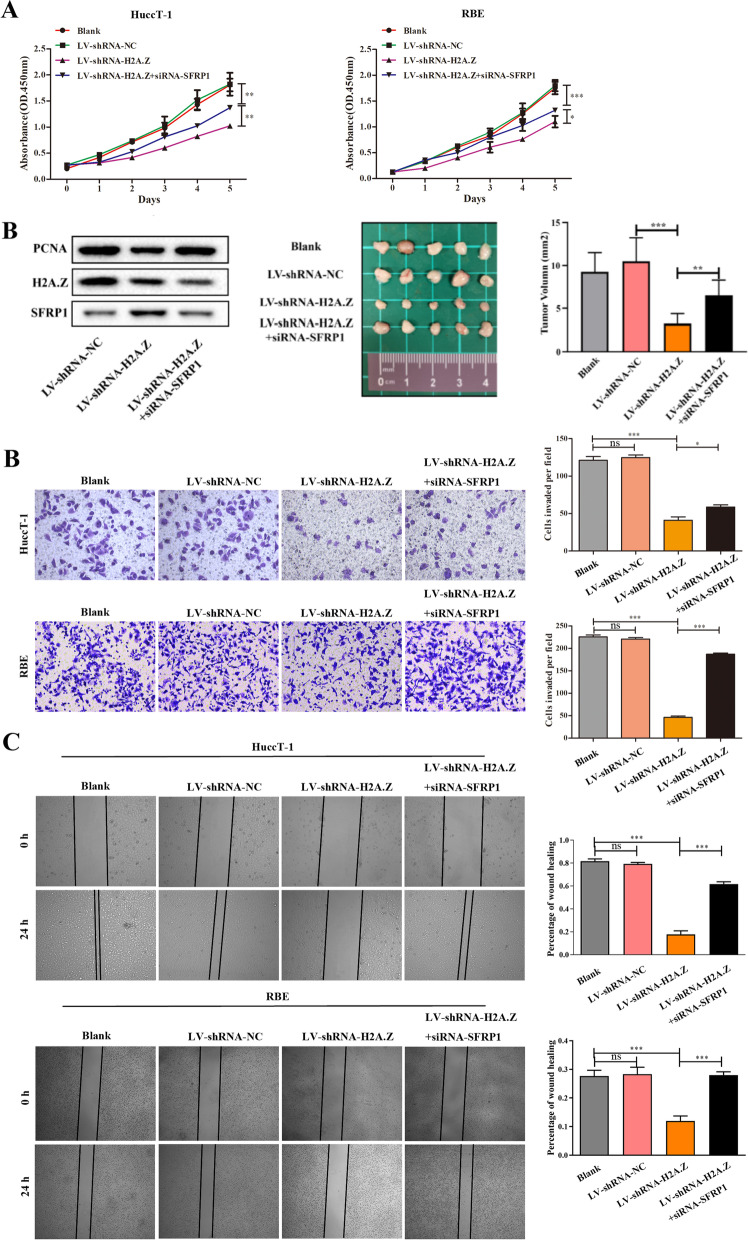
The effect of H2A.Z and/or SFRP1 silencing on the proliferation and invasion of ICC cells. HuccT-1 and RBE cells were transduced with LV-shRNA-NC (negative control), LV-shR-H2A.Z and/or siR-SFRP1 to generate cells with silencing. Proliferation, invasion and wound healing were evaluated. **A** The dynamic proliferation of ICC cells. **B** The result of nude mouse tumorigenicity assay, and the expression of PCNA in mouse tissues. **C** The invasion of ICC cells (200 × magnification). **D** The wound healing ability of ICC cells. The data shown are representative images or expressed as individual mean values or the means ± SDs of each group in three separate experiments. *p < 0.05, **p < 0.01, ***p < 0.001

Since H2A. Z downregulated SFRP1 expression, we studied the effect of H2A.Z and/or SFRP1 silencing on the proliferation and invasion of ICC cells. In comparison with that of the control cells, H2A.Z silencing strikingly inhibited the proliferation of HuccT-1 and RBE cells, and this effect was significantly mitigated by simultaneous silencing of SFRP1 in both cell lines (Fig. 5A). In contrast, SFRP1 silencing alone only moderately reduced proliferation in both cell lines. Then, we performed a tumorigenicity assay in nude mice (Fig. 5B). We found that the volumes of tumours in the H2A.Z-silenced group were significantly lower than those in the normal control group. SFRP1 silencing partially rescued the inhibited proliferation of ICC cells. In mouse tissues, the expression of proliferating cell nuclear antigen (PCNA), a proliferation indicator, was consistent with the results of the tumorigenicity assay in nude mice. Accordingly, we hypothesized that simultaneous silencing of H2A.Z and SFRP1 may promote cell proliferation via unknown mechanisms. Similar patterns were observed among the different groups of cells in the wound healing and Transwell invasion assays (Fig. 5C and D). These data demonstrated that H2A.Z silencing inhibited the proliferation and invasion of ICC cells and that these effects were mitigated by SFRP1 silencing in vitro.

## Discussion

**Fig. 6:**
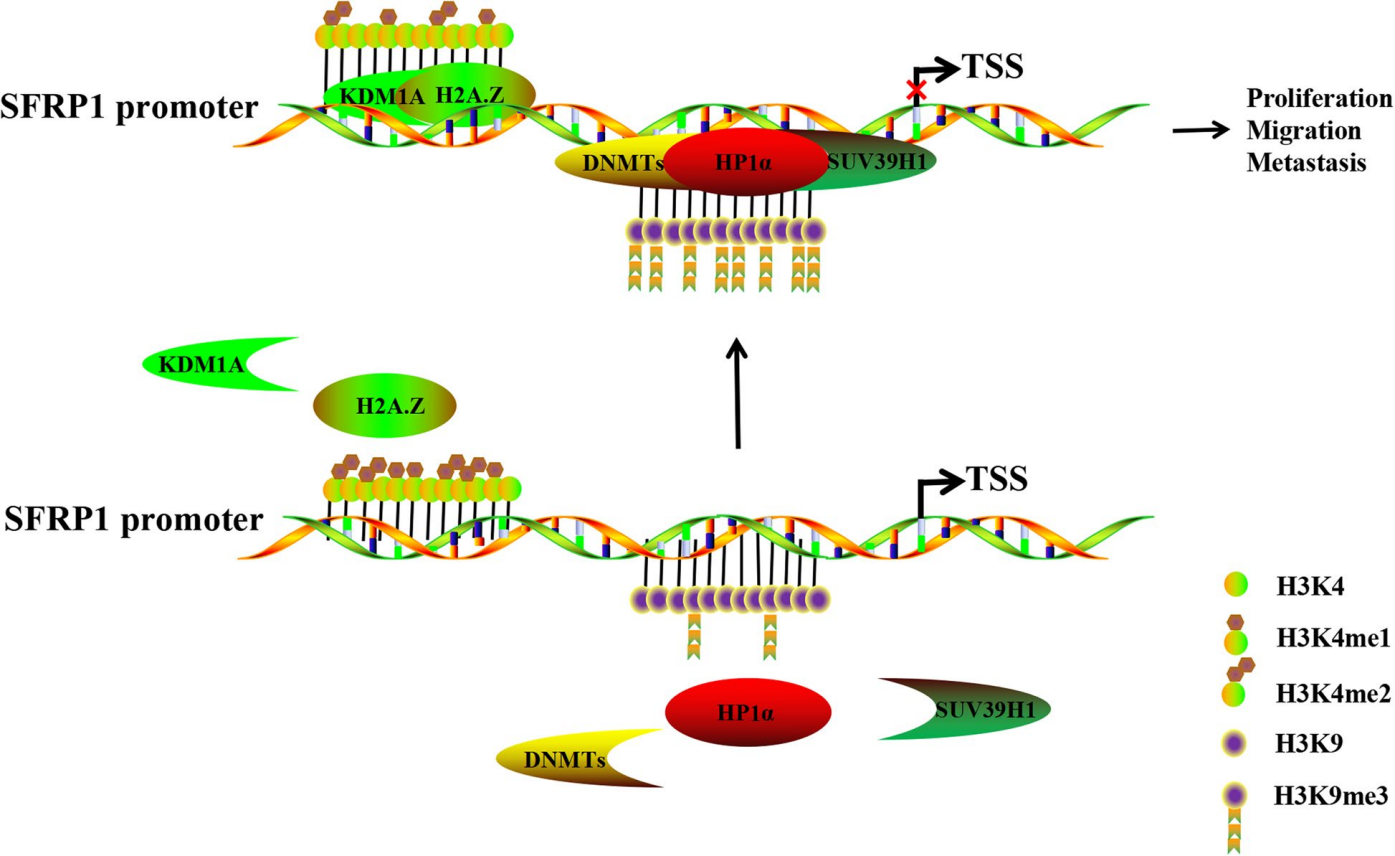
Schematic illustration of the role of H2A.Z in regulating SFRP1 promoter activity in ICC. In the nucleus, H2A.Z recruits KDM1A, H3K4me1 and H3K4me2 to form a complex to promote demethylation upstream of the SFRP1 promoter and coordinates with HP1α, which is located at downstream CpG islands and recruits SUV39H1/H3K9me3/DNMTs to repress SFRP1 transcription

Over the past three decades, the histone variant H2A.Z has been the subject of intense study, revealing not only the enzymatic activities required for its chromatin deposition but also the interconnected posttranslational regulatory mechanisms as well as its dynamics in response to signaling pathways. H2A.Z has been shown to be associated with diverse biological processes, including memory [33–35] and epithelial-to-mesenchymal transition (EMT) [36]. At the molecular level, it has been linked to heterochromatin regulation [37–39], an anti-silencing function at yeast chromatin boundaries [40, 41], DNA repair [42, 43], and transcriptional regulation [44, 45]. However, it is unclear how H2A.Z regulates such a wide spectrum of different processes, particularly in ICC. Our previous study demonstrated that SFRP1 regulates the occurrence and development of cholangiocarcinoma. In short, as Fig. 6 shows, HP1α can recruit proteins such as SUV39H1, H3K9me3, and DNMTs and lead to the hypermethylation of the secreted frizzled-related protein 1 (SFRP1) promoter, which suppresses the expression of SFRP1 [46, 47]. In the past, it has been claimed that H2A.Z is primarily found at the transcription start sites (TSSs) of genes. In yeast, H2A.Z is highly enriched at the TSSs of both active and inactive genes [48]. Its occupancy at the TSS is negatively correlated with the expression of genes: H2A.Z occupancy is more pronounced at the TSSs of poorly expressed genes than at those of induced genes [49, 50]. In subsequent studies, it became increasingly clear that H2A.Z is not only found exclusively at TSSs but can also be detected at other regulatory elements, such as enhancers and insulators, although to a lesser extent in several different species [51, 52]. A further indication that H2A.Z is an enhancer mark is the fact that it is enriched at a p53-binding site located around 2.2 kb upstream of the TSS of the human p21 gene [53].

Based on our previous study, in this study, we explored the importance of SFRP1 and H2A.Z in the development and progression of ICC. We first found that SFRP1 and H2A.Z were expressed predominantly in the nucleus in cells in ICC tissues and that downregulated SFRP1 expression was associated with upregulated H2A.Z expression in ICC tissues and was also associated with pathological grade, lymph node metastasis and distant metastasis. In addition, high expression of H2A.Z or low expression of SFRP1 was related to poor survival in ICC patients. The significant association with poor survival suggested that low SFRP1 and high H2A.Z expression may be a new biomarker for prognosis in ICC patients.

To understand the regulatory role of H2A.Z in SFRP1 expression, we characterized the relative levels of H2A.Z expression in ICC and nontumor HIBEpic cells and found that the levels of H2A.Z expression in ICC cells was significantly higher than that in nontumor HIBEpic cells. Furthermore, H2A.Z silencing enhanced SFRP1 expression in HuccT-1 and RBE cells. Then, we also found that the levels of SFRP1 expression were lower in ICC cells than in HIBEpic cells. We found no apparent differences in expression between ICC and nontumor HIBEpic cells with prior SFRP1 overexpression and control cells. These results demonstrated that H2A.Z might regulate SFRP1 expression unidirectionally. Then, we also found that H2A.Z was mainly localized in the nucleus in ICC cells but showed no apparent difference in nontumor HIBEpic cells. To explore the possible mechanisms, we used the STRING database and, interestingly, found that H2A.Z might recruit the demethylase KDM1A to form a complex to regulate the expression of related genes via demethylation of H3K4. To verify this hypothesis, we used a ChIP assay and found that H2A.Z enrichment was especially high at the P3 region upstream of the SFRP1 promoter, the -151 ~ -136 bp region upstream of the SFRP1 transcription start site. Using coimmunoprecipitation, we also found that H2A.Z can form a complex with KDM1A, H3K4me1, and H3K4me2 in HuccT-1 and RBE cells. In addition, through a ChIP assay, we found that the enrichment of KDM1A was significantly higher in the P3 region upstream of the SFRP1 promoter in ICC cells, while the enrichment of H3K4me1 and H3K4me2 was lower. This evidence indicated that H2A.Z-KDM1A might bind upstream of the SFRP1 transcription start site to repress SFRP1 expression via demethylation of the P3 region.

In our previous study, HP1α was found to inhibit SFRP1 expression by recruiting proteins such as SUV39H1, H3K9me3, and DNMTs and promoting DNA methylation of the SFRP1 promoter [35, 47]. To further explore the essential associations, we also performed a ChIP assay to test whether HP1α specifically binds to the P1 region of the SFRP1 promoter, the -116 ~  + 1 bp region upstream of the SFRP1 transcription start site. BSP also revealed that H2A.Z silencing significantly increased the relative methylation level in the SFRP1 P3 region in HuccT-1 and RBE cells. Moreover, induction of H2A. Z overexpression significantly decreased SFRP1 promoter-driven luciferase activity in ICC and HEK293T cells. These data indicated that H2A.Z binds to the SFRP1 promoter and recruited KDM1A, H3K4me1 and H3K4me2 to the P3 region upstream of the SFRP1 promoter to promote its demethylation and acts as an enhancer to increase the methylation level in the downstream P1 region via the HP1α/SUV39H1/H3K9me3/DNMT complex. As a result, enhancer demethylation and downstream methylation synergistically promote the inhibition of SFRP1 expression.

To the best of our knowledge, this is the first demonstration that H2A.Z is a negative regulator of SFRP1 expression during the development and progression of ICC. These findings might shed fresh perspectives on the occurrence of ICC. We found that H2A.Z silencing significantly attenuated the proliferation, wound healing capacity and invasion of HuccT-1 and RBE cells in vitro. These inhibitory effects of H2A.Z silencing were alleviated by concomitant silencing of SFRP1, whereas silencing SFRP1 alone only moderately attenuated the proliferation, wound healing capacity and invasion of HuccT-1 and RBE cells. These data suggest that H2A.Z may promote the expression of oncogenic proteins, leading to the progression of ICC.

## Conclusions

Our data indicated that downregulation of SFRP1 expression is associated with upregulation of H2A.Z expression in ICC tissues and poor survival in patients with ICC. H2A.Z bound to the P3 region of the SFRP1 promoter and interacted with the KDM1A/H3K4me1/H3K4me2 complex to promote the demethylation and reduce the expression of SFRP1 in ICC cells. H2A.Z positively regulated the proliferation and invasion of ICC cells, and these effects were mitigated by SFRP1 expression. Hence, our findings indicated that H2A.Z overexpression is a biomarker for the prognosis of ICC and suggested that H2A.Z may be a therapeutic target for intervention in ICC. 

## Supplementary Information

**Additional file 1:** **Fig. S1.** Analysis of the expression data from GEO database. The difference of the expression of H2A.Z and SFRP1 between ICC tissue and normal bile duct was visualized by box plot. (**A**) GSE32879. (**B**) GSE76297. **p<0.01, ***p<0.001.

**Additional file 2:** **Fig. S2.** The site of H2A.Z or HP1α binds to SFRP1 promoter. (**A**) The ChIP assay analysis of H2A.Z or HP1α enrichment in the SFRP1 promoter in HEK293T cells. IgG served as a negative control. Relative enrichment fold=[%(ChIP/Input)]/[%(IgG/Input)]. (**B**) Dual luciferase assays demonstrate that H2A.Z attenuates the SFRP1 promoter-controlled luciferase activity in HEK293T and ICC cells (pDNA: empty plasmid vector; pGL3: empty pGL3 plasmid vector; pDNA-H2A.Z: pcDNA-H2A.Z plasmid; SFRP1-pGL3: The SFRP1 promoter inserted in the pGL3 plasmid). Data are expressed as individual mean values or the means ± SD of each group from three separate experiments. *P<0.05, **p<0.01, ***p<0.001.

**Additional file 3:** **Fig. S3.** Analysis of the methylation data from GEO database. The difference of the methylation level of H2A.Z and SFRP1 between ICC tissue and normal bile duct was visualized by box plot. (**A**) GSE38860. (**B**) GSE49656. *P<0.05, **p<0.01, ***p<0.001.

**Additional file 4:** **Table S1.** Detailed information of the microarray used in this study.

**Additional file 5.** Supplement.

## Data Availability

The microarrays analyzed in the current study are available in GEO database (https://www.ncbi.nlm.nih.gov/geo/) (24). The datasets analyzed during the current study are available from the corresponding author on reasonable request.

## Abbreviations

BSP: Bisulfite sequencing PCR
CCK-8: Cell Counting Kit-8
ChIP: Chromatin immunoprecipitation
Co-IP: Co-immunoprecipitation
ECL: Enhanced chemiluminescence
EMT: Epithelial-to-mesenchymal transition
FBS: Fetal bovine serum
H3K4me1/2: Mono- and di-methylation of histone H3 at lysine 4
H3K9me3: Trimethylated Lys9 of histone H3
ICC: Intrahepatic cholangiocarcinoma
HRP: Horseradish peroxidase
IHC: Immunohistochemistry
OD: Optical density
PTM: Post-translational modification
PVDF: Polyvinylidene fluoride
SDS-PAGE: Sodium dodecyl sulfate polyacrylamide gel electrophoresis
SEM: Standard error of mean
SFRP1: Secreted frizzled-related protein 1
TSS: Transcriptional start site
PCNA: Proliferating cell nuclear antigen
