# Supplementary figures and images for: The H2A.Z-KDM1A complex promotes tumorigenesis by localizing in the nucleus to promote SFRP1 promoter methylation in cholangiocarcinoma cells
Source: BMC Cancer. 2022 Nov 11;22:1166. doi: 10.1186/s12885-022-10279-y (PMC9652970; doi:10.1186/s12885-022-10279-y)

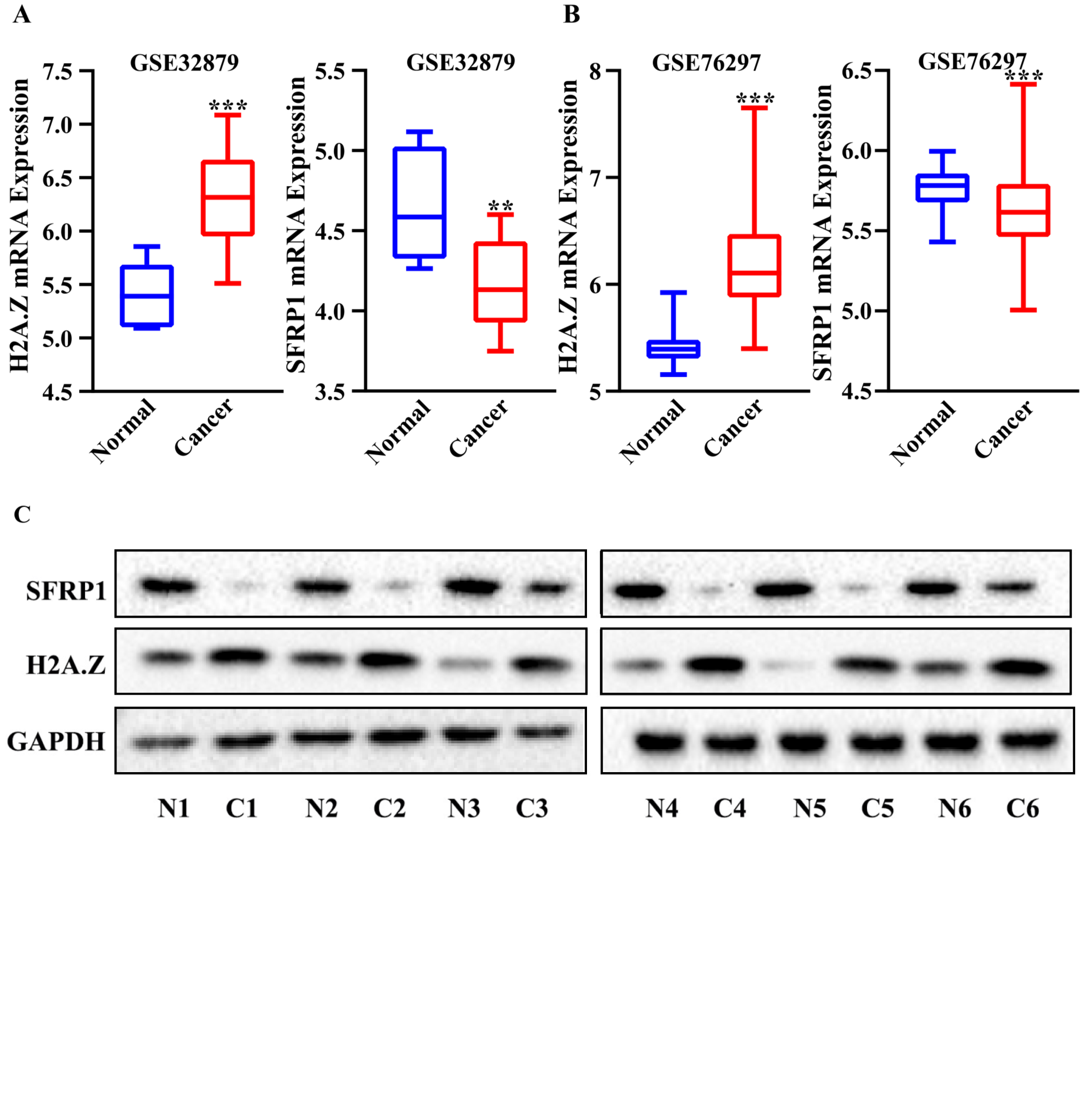

Supplement: Supplementary file 1 — Additional file 1: Fig. S1. Analysis of the expression data from GEO database. The difference of the expression of H2A.Z and SFRP1 between ICC tissue and normal bile duct was visualized by box plot. (A) GSE32879. (B) GSE76297. **p<0.01, ***p<0.001. [file 12885_2022_10279_MOESM1_ESM.tif]

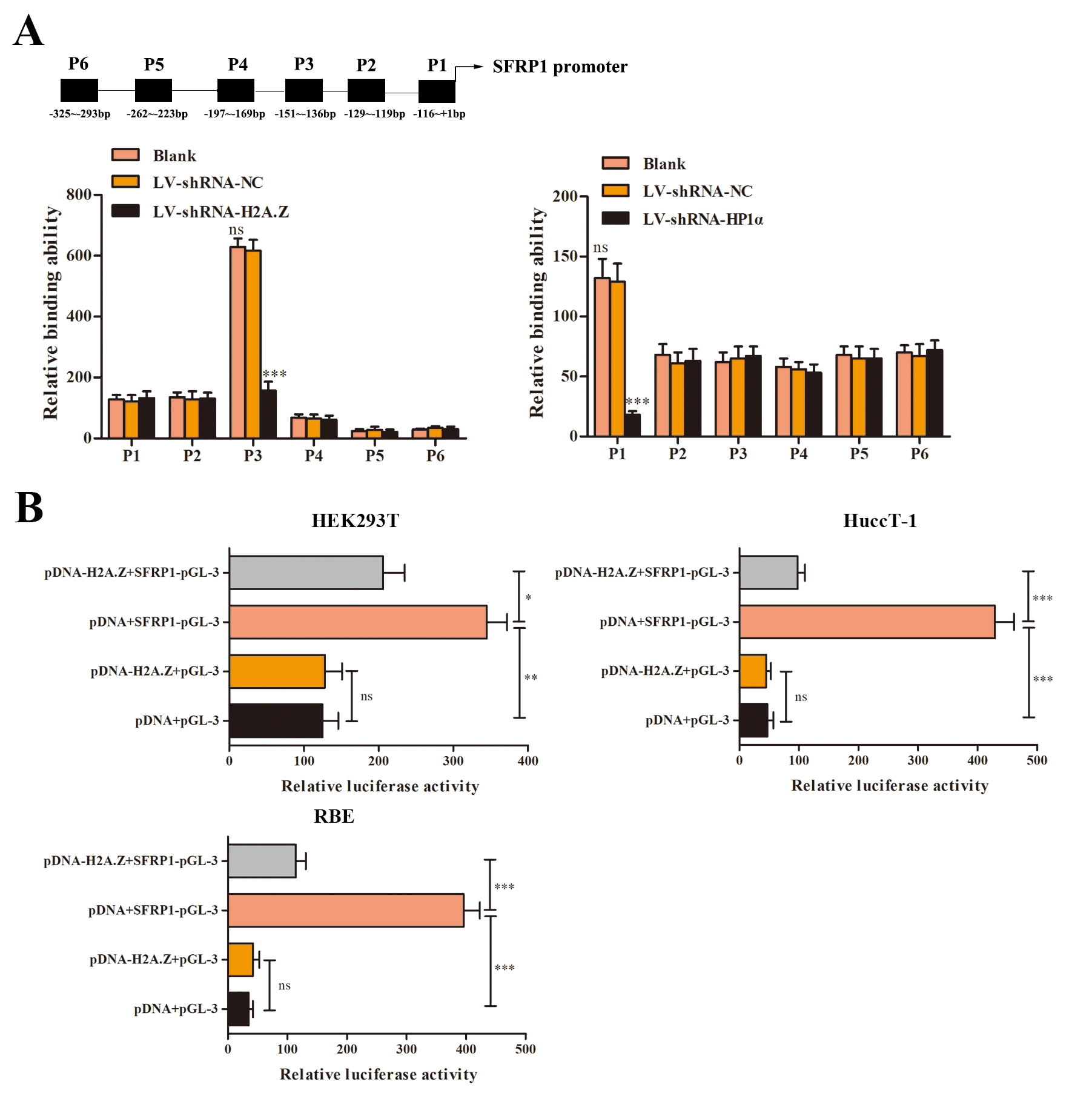

Supplement: Supplementary file 2 — Additional file 2: Fig. S2. The site of H2A.Z or HP1α binds to SFRP1 promoter. (A) The ChIP assay analysis of H2A.Z or HP1α enrichment in the SFRP1 promoter in HEK293T cells. IgG served as a negative control. Relative enrichment fold=[%(ChIP/Input)]/[%(IgG/Input)]. (B) Dual luciferase assays demonstrate that H2A.Z attenuates the SFRP1 promoter-controlled luciferase activity in HEK293T and ICC cells (pDNA: empty plasmid vector; pGL3: empty pGL3 plasmid vector; pDNA-H2A.Z: pcDNA-H2A.Z plasmid; SFRP1-pGL3: The SFRP1 promoter inserted in the pGL3 plasmid). Data are expressed as individual mean values or the means ± SD of each group from three separate experiments. *P<0.05, **p<0.01, ***p<0.001. [file 12885_2022_10279_MOESM2_ESM.tif]

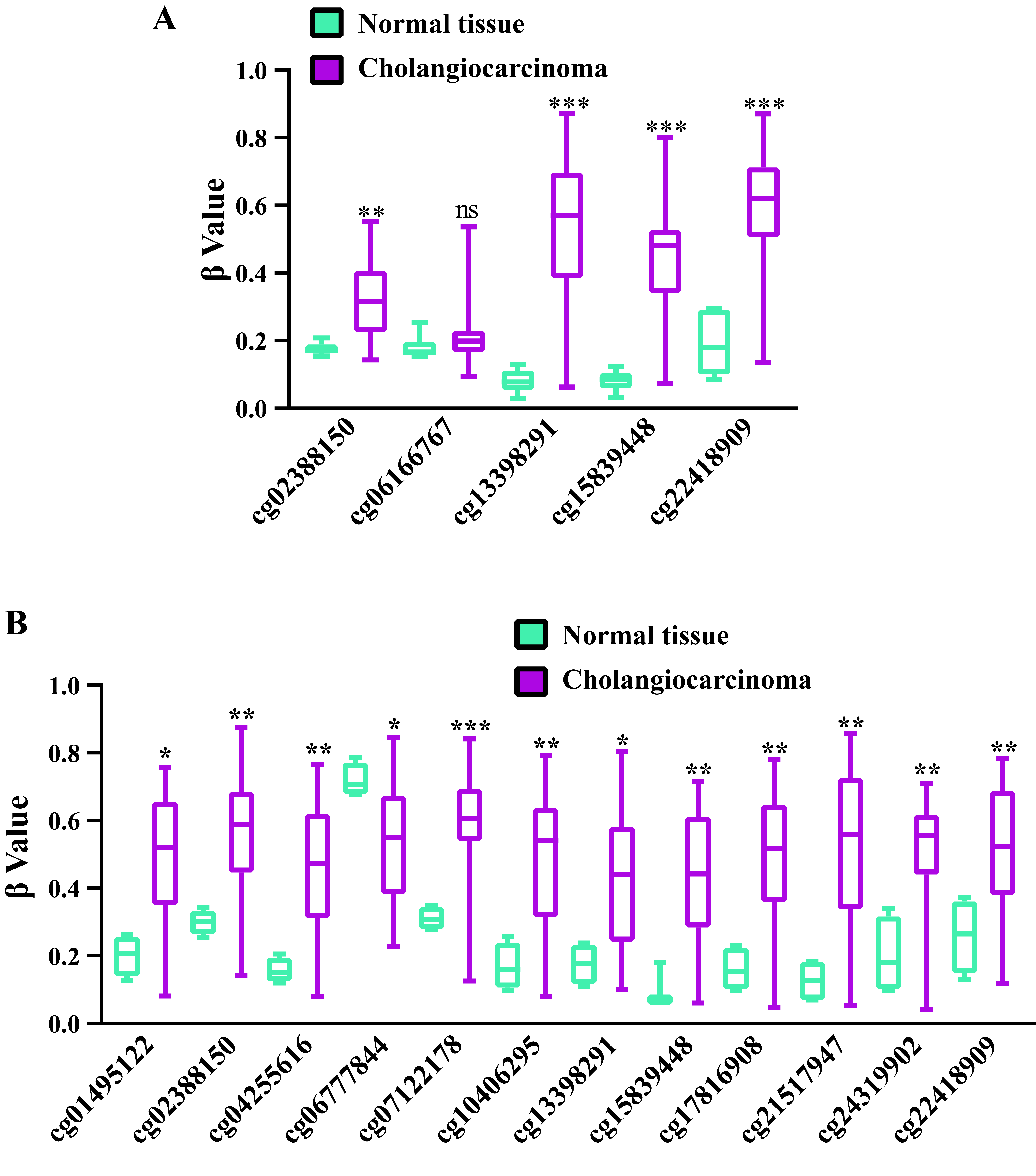

Supplement: Supplementary file 3 — Additional file 3: Fig. S3. Analysis of the methylation data from GEO database. The difference of the methylation level of H2A.Z and SFRP1 between ICC tissue and normal bile duct was visualized by box plot. (A) GSE38860. (B) GSE49656. *P<0.05, **p<0.01, ***p<0.001. [file 12885_2022_10279_MOESM3_ESM.tif]
