# Supplementary material for: The H2A.Z-KDM1A complex promotes tumorigenesis by localizing in the nucleus to promote SFRP1 promoter methylation in cholangiocarcinoma cells
Source: BMC Cancer. 2022 Nov 11;22:1166. doi: 10.1186/s12885-022-10279-y (PMC9652970; doi:10.1186/s12885-022-10279-y)

Figure2

2A

H2A.Z

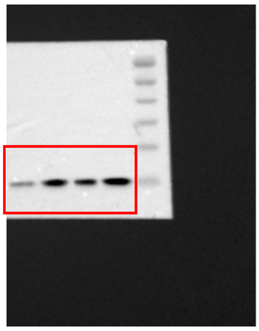

GAPDH

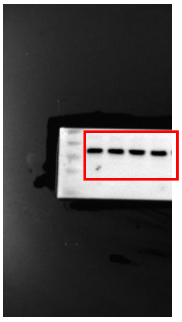

2B

SFRP1

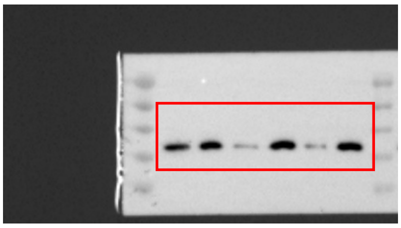

GAPDH

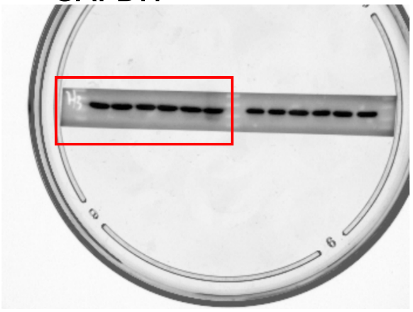

2C

SFRP1

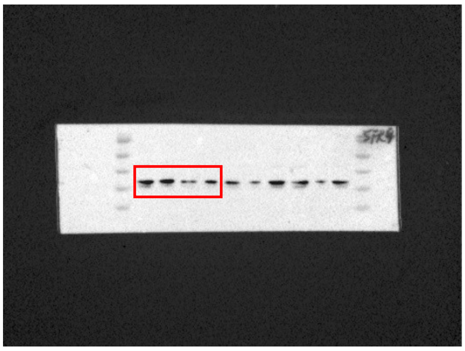

GAPDH

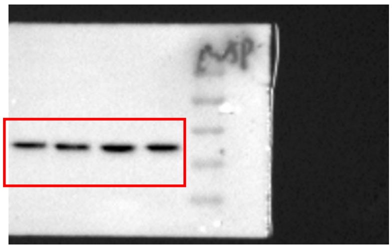

2D

H2A.Z

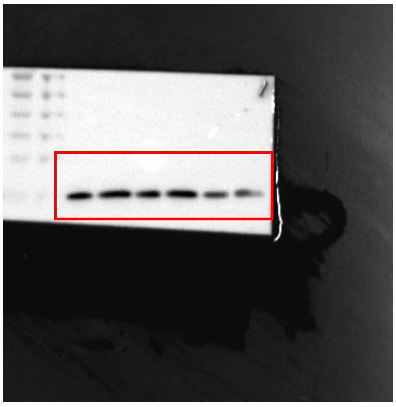

GAPDH

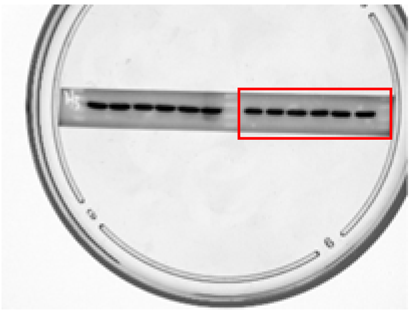

Figure3

3A

H2A.Z

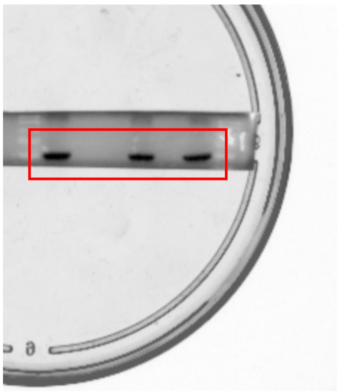

H3

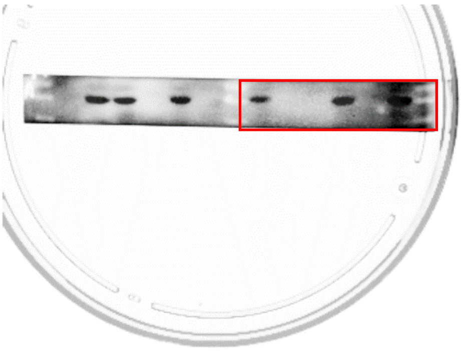

GAPDH

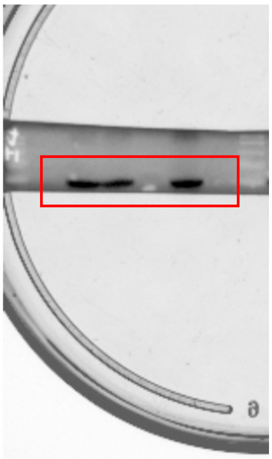

3C

H2A.Z-HIBEpic

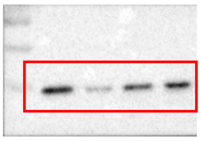

H2A.Z-HuccT1 and RBE

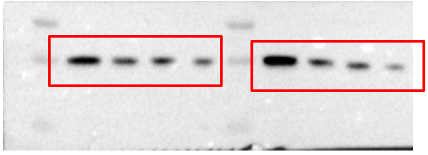

3E

IP-KDM1A:

HuccT-1

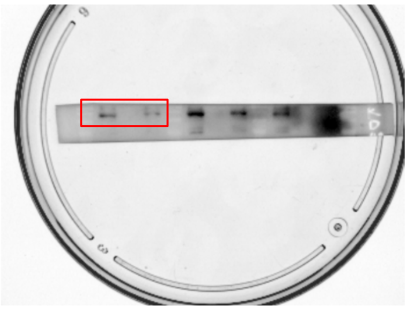

RBE and HIBEpic

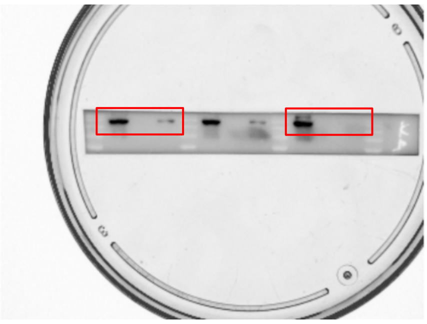

IP-H3K4me1

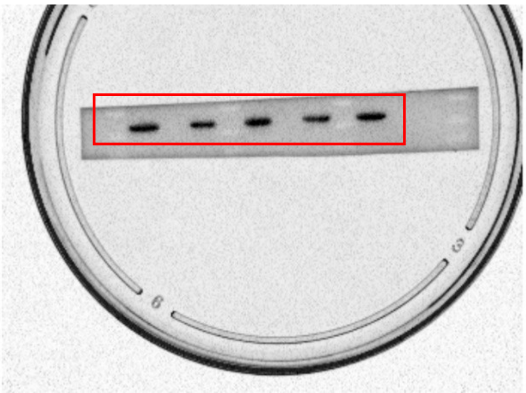

IP-H3K4me2

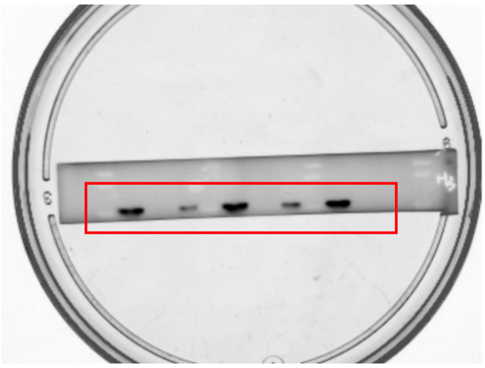

IP-H2A.Z

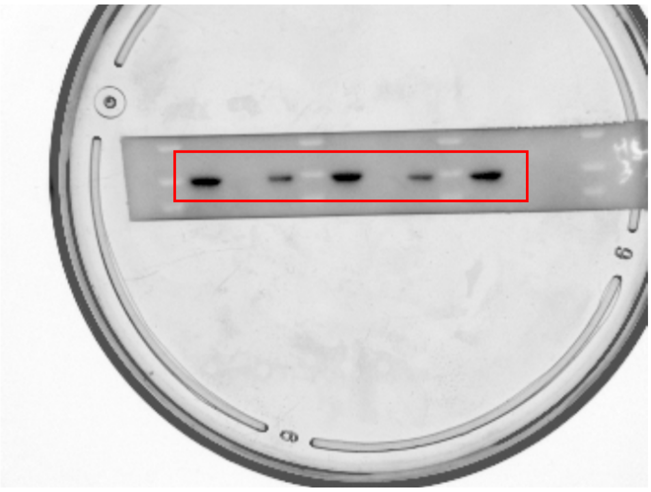

3F SFRP1

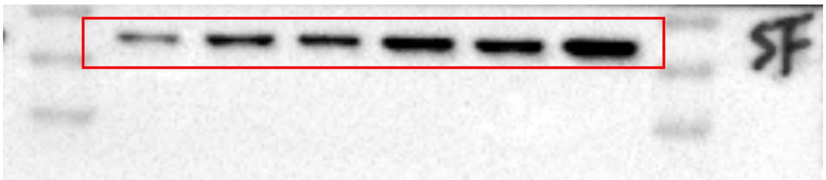

3F GAPDH

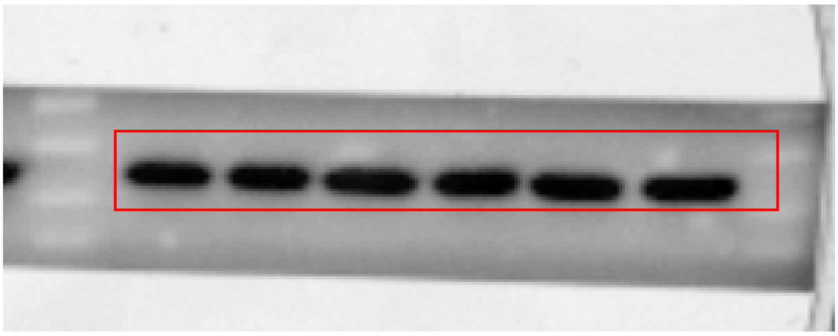

Figure 5

5B

PCNA

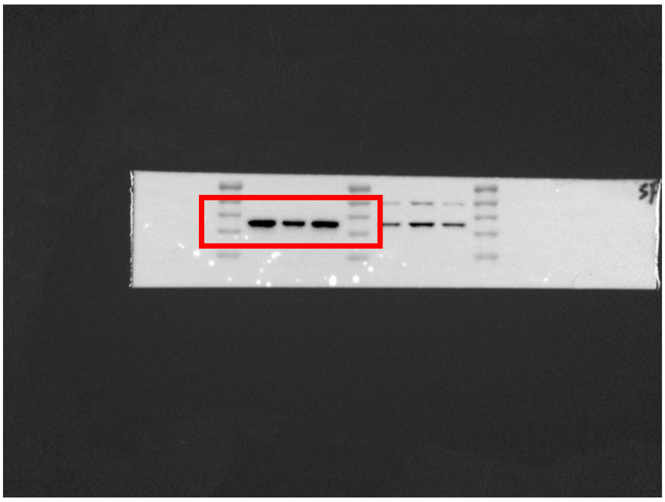

H2A.Z

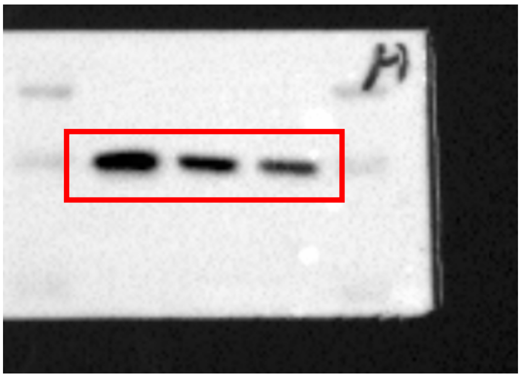

SFRP1

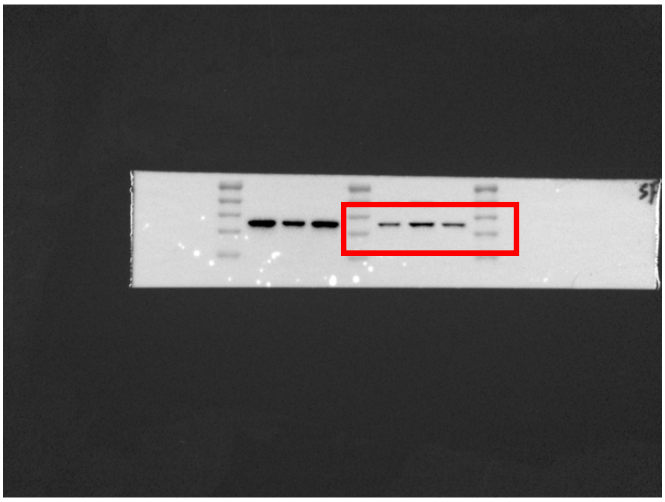

Figure S1C

H2A.Z

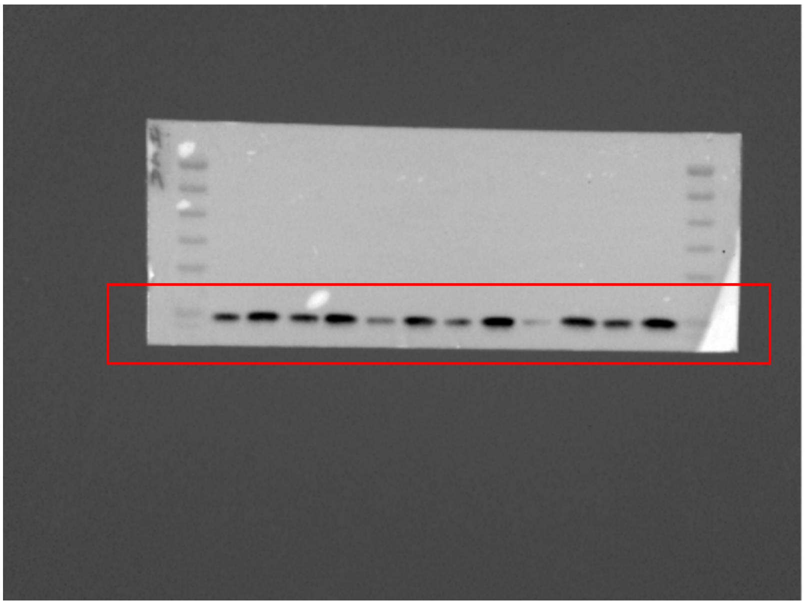

SFRP1

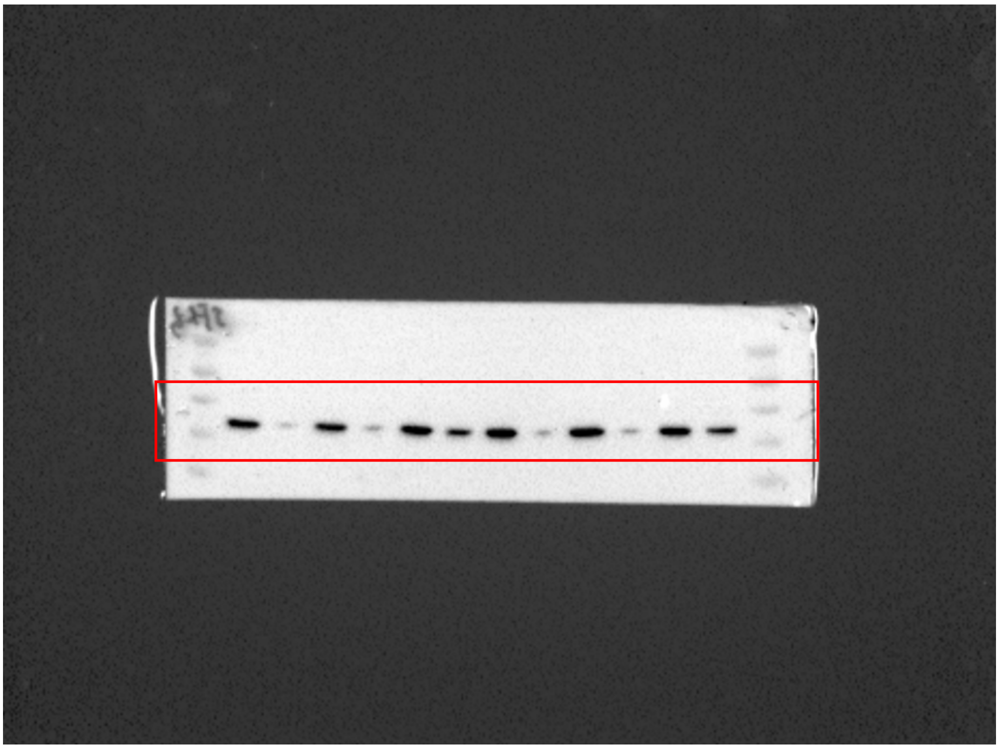

GAPDH

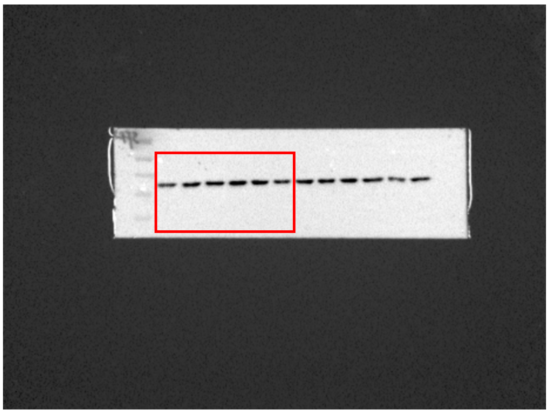

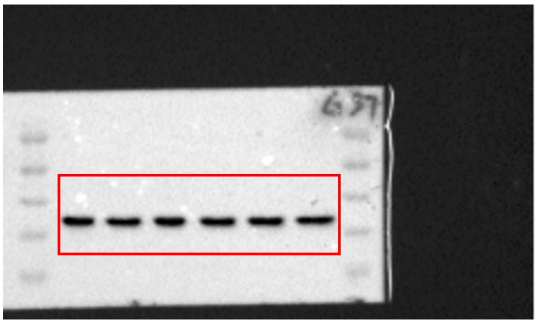

Supplement: Supplementary file 5 — Additional file 5. Supplement. [file 12885_2022_10279_MOESM5_ESM.pdf]
